# Challenges with social distancing during the COVID-19 pandemic among Hispanics in New York City: a qualitative study

**DOI:** 10.1186/s12889-021-11939-7

**Published:** 2021-10-26

**Authors:** Christopher J. Gonzalez, Bryan Aristega Almeida, George S. Corpuz, Hector A. Mora, Oluwatobi Aladesuru, Martin F. Shapiro, Madeline R. Sterling

**Affiliations:** Department of Medicine, Weill Cornell Medical College, Cornell University, 525 E 68th St., New York, NY 10065 USA

**Keywords:** Hispanic, Latino, Disparities, Equity, Social distancing, Quarantining, COVID-19, Social determinants, Crowding, Essential workers

## Abstract

**Background:**

Hispanics in the United States are disproportionately affected by the novel coronavirus (COVID-19). While social distancing and quarantining are effective methods to reduce its spread, Hispanics, who are more likely to be essential workers and live in multigenerational homes than non-Hispanics, may face challenges that limit their ability to carry out these preventative efforts. We elicited the experiences of Hispanic adults with social distancing and self-quarantining during the COVID-19 pandemic in New York.

**Methods:**

In this qualitative study, Hispanic adults receiving care at a federally qualified community health center in East Harlem, New York, were recruited for remote one-on-one semi-structured interviews from 5/15/2020 to 11/17/2020. Interviews were conducted by a bilingual interviewer in Spanish or English, using a semi-structured topic guide informed by the Health Belief Model. Audio-recordings were professionally transcribed. We used thematic analysis to iteratively code the data. Each transcript was independently coded by two research team members, then reconciled by a third. Major themes and subthemes were identified.

**Results:**

Among 20 participants, four major themes emerged; Hispanics were: (1) fearful of contracting and transmitting COVID-19, (2) engaging in practices to reduce transmission of COVID-19, (3) experiencing barriers to social distancing and quarantining, and (4) facing an enduring psychological and physical toll from COVID-19.

**Conclusions:**

Despite understanding the risks for contracting COVID-19 and taking appropriate precautions, Hispanics faced numerous challenges to social distancing and quarantining, such as living in crowded, multi-generational households, working as essential workers, and providing unpaid care to family members. Such challenges took a toll on their physical, emotional, and financial well-being. Our findings suggest that a tailored approach to public health messaging and interventions for pandemic planning are warranted among members of this community. Further research is needed to understand and mitigate the long term physical and psychological consequences of the pandemic among Hispanics.

**Supplementary Information:**

The online version contains supplementary material available at 10.1186/s12889-021-11939-7.

## Background

Hispanics are the largest ethno-racial minority group in the United States (US) and have been disproportionately affected by the novel coronavirus (COVID-19) [1]. By March 2021, age-adjusted mortality rates in New York City, one of the primary epicenters of the pandemic, were 324/100,000 for Hispanics compared to 173/100,000 for non-Hispanic Whites [2, 3]. Neighborhood-level analyses have revealed that mortality rates per capita were highest in the Bronx, a borough that is nearly 50% Hispanic [2, 4, 5]. Additionally, communities with higher population shares of Hispanic residents have had higher rates of confirmed COVID-19 cases [6, 7].

A key strategy to reduce the transmission of COVID-19 has been through social distancing, which involves increasing the physical space (6 feet) between people to avoid spreading illness [8]. This, however, may be challenging in predominantly Hispanic communities, since many Hispanics are frontline workers in the food, cleaning and healthcare industries [9, 10]. As such, social distancing is not always possible. Quarantining at home may also pose particular challenges for Hispanics since many live in crowded multigenerational homes, which have been associated with unfavorable outcomes [11–13]. Despite these barriers, few studies have examined the psychosocial, socioeconomic and logistic factors which affect the understanding and the feasibility of social distancing among Hispanics.

In this context, we aimed to understand the experiences of an under-resourced Hispanic community with social distancing and quarantining during the COVID-19 pandemic in New York City. Specifically, we sought to elucidate unique drivers of and challenges with social distancing and quarantining among Hispanic patients of a federally qualified health center.

## Methods

### Setting and study population

This qualitative study was conducted from May 15 to November 17, 2020 in collaboration with a federally-qualified community health center that serves a predominantly Hispanic community [14]. The health center is a primary care clinic that was established in East Harlem over 40 years ago and provides numerous resources, including immigration and legal services, to the surrounding community. Due to its location and proximity to the boroughs of Queens and the Bronx, the clinic serves a diverse Hispanic population. One study author (CJG) is a clinician at this community health center.

Patients were eligible to participate if they were: 1) 18 years of age or older; 2) self-identified as Hispanic; and 3) spoke English or Spanish. Convenience sampling was used initially to recruit participants. The investigators transitioned to purposive sampling to achieve a sample that was balanced across age, gender, prior confirmed or probable exposure to COVID-19, and English language proficiency [15]. Eligible and interested participants were first identified by the primary care providers at the clinic. Next, using a standardized script, the lead investigator (CJG) directly contacted interested participants by telephone to further assess eligibility and explain the details of this voluntary study. Participants provided verbal consent, including permission to record the interview and publish de-identified excerpts from the interview. Following the interview, they received a $25 gift card. The study was approved by the Weill Cornell Medicine Institutional Review Board (Protocol 20–04021971). This manuscript adheres to the Consolidated Criteria for Reporting Qualitative Research (COREQ) reporting guideline [16].

### Data collection

One researcher (CJG) trained in qualitative methods conducted interviews in English and Spanish using a semi-structured topic guide and Zoom software. The topic guide was informed by the Health Belief Model, [17] a conceptual model that often is used in health services research to understand public and patient engagement in specific health behaviors. This model includes four main constructs which predict health behavior: 1) perceived threat from a health condition (comprised of perceived susceptibility and perceived severity); 2) likelihood of enacting the health behavior (comprised of perceived benefits and perceived barriers); 3) cues to action (motivating factors); and 4) self-efficacy (one’s belief in the ability to actually perform the change). With these four guiding constructs, the topic guide interview questions broadly focused on: 1) understanding and perceived risk of COVID-19, 2) perceived benefits of social distancing measures, 3) barriers and challenges to effectively socially distance and quarantine, and 4) potentially helpful resources to facilitate social distancing and quarantine practices (Additional File 1). The topic guide was reviewed and edited for clarity by senior authors (MRS, MFS) and by leadership at the community health center. The topic guide then was translated into Spanish and reviewed by multiple native Spanish speakers for clarity and validity.

Interviews lasted between 20 and 30 min and were audio recorded and professionally translated and transcribed by Ubiqus [18]. Interviews were not repeated and transcripts were not returned to participants for comment or correction. Interviews continued until no new themes emerged [19]. In addition to the qualitative components of the interviews, we collected self-reported demographic data including: age, gender, race/ethnicity, Hispanic origin, nativity (US-born vs not), English proficiency, education, medical history, household size, and health literacy.

### Analysis

We performed a thematic analysis of the data, an approach that has been widely used in health-related research when an existing conceptual framework is utilized [20]. Data were managed, organized, and analyzed using Dedoose, a protected qualitative software system [21]. Two investigators (OA, HAM) used an open coding system to independently code an initial transcript. Coding of this initial transcript was then reconciled by the lead investigator (CJG) and codes were consolidated into an initial code book that was then reviewed by a senior author (MRS). This consolidated code book was used by two coders at a time (BAA, GC, HAM, OA) to individually analyze subsequent transcripts, adding new codes as they emerged. All coding was reconciled and new codes approved by the lead authors (CJG and MRS). The final codebook consisted of 65 unique codes (Additional File 2). Saturation, the point at which no new codes emerged, was achieved at interview 16 [22]. We conducted 4 additional interviews beyond saturation because these participants were already scheduled to participate. An iterative coding process was used to deductively and inductively label data according to repeated concepts [23, 24]. Once coding of all interviews was completed, these codes were consolidated into categories according to similar and contrasting properties and dimensions, and then consolidated into unifying themes, reconciling discrepancies through discussion [25].

## Results

A total of 20 participants were interviewed (Table 1). Participants had a mean (SD) age of 47.6 (17.0) years, 65% were female, 65% were not born in the United States, and 70% had at least completed high school. Most identified as Puerto Rican (40%), Mexican (35%), and/or Ecuadorian (30%). A total of 20% of participants had two or more underlying medical conditions, the most common being high blood pressure (*n* = 7), heart disease (*n* = 4), and diabetes (*n* = 3).

**Table 1:** Demographic characteristics of 20 participants

	n (%) or mean (SD)
Interview language, Spanish	12 (60%)
Age, years	47.6 (17.0)
Female gender	13 (65%)
Hispanic Origin^a^	
Puerto Rico	8 (40%)
Mexico	7 (35%)
Ecuador	6 (30%)
Cuba	1 (5%)
El Salvador	1 (5%)
Colombia	1 (5%)
Nativity, Immigrant	13 (65%)
English Proficiency	
Well or Very well	13 (65%)
Not well or not at all	7 (35%)
Educational attainment	
Did not complete high school	9 (45%)
Completed high school	5 (25%)
Some college	3 (15%)
Completed college	3 (15%)
Number of comorbidities^b^	
0	5 (25%)
1	11 (55%)
2	2 (10%)
≥ 3	2 (10%)
Current smoker	3 (15%)
Household size	
1 to 2 people	6 (30%)
3 to 4 people	9 (45%)
≥ 5 people	5 (25%)
Health literacy^c^	
Extremely or quite a bit	16 (80%)
Somewhat	3 (15%)
A little bit or not at all	1 (5%)

^a^Participants could select multiple Hispanic origin identities

^b^Comorbidities were based on self-reported history of diabetes, high blood pressure, high cholesterol, any history of cancer, heart problems, liver problems, kidney problems, lung problems, problems with the immune system, or depression/anxiety

^c^Based on the question “How confident are you filling out medical forms by yourself?”

Four major themes emerged, each with subthemes (Table 2). We present them along with representative quotations.

**Table 2:** Major Themes and Subthemes Summarizing Experiences with Social Distancing and Self-Quarantining Among Hispanics During the Coronavirus Disease 2019 (COVID-19) Pandemic

(1) Fear of contracting and transmitting COVID-19 • Aware and afraid • Frequent exposure • Transmission to family and others • Risk perception	
(2) Engaging in practices to reduce transmission of COVID-19 • Standard precautions • Gathering information • Extraordinary precautions	
(3) Barriers to social distancing and quarantining • Social and financial obligations • Accessing basic goods and services • Unreliable information	
(4) Enduring psychological and physical toll of the pandemic • Psychological impact • Physical health • Financial Strain • Mechanisms of hope and coping	

### Theme (1): fear of contracting and transmitting COVID-19

Participants were acutely aware of COVID-19. They reported hearing about its spread through news updates on television and received frequent reminders about the importance of social distancing in various settings. In addition, many participants had relatives or friends who had contracted or died from COVID-19, which compounded their awareness and their fear of it. As one participant summarized it:

*‘We began to see it in people close to us. My friend was admitted to the hospital, then another person passed away. Then my son’s coach also passed away.’*

Beyond their concerns about contracting COVID19 themselves, many worried about unknowingly transmitting the virus to their family members. One participant explained:

*‘I was working during the pandemic and I was really, really scared for my mother and my grandson, because I didn’t want to infect them.’*

Participants were particularly concerned about contracting and transmitting COVID-19 among those whom they perceived to be high risk. This included older adults and people with underlying medical conditions. As one participant expressed:

*‘My wife has diabetes. She would die if she got infected.’*

### Theme (2): engaging in practices to reduce transmission of COVID-19

As fears and concerns about COVID-19 arose, participants reported taking numerous precautions to minimize their risk. They described attempts to stay indoors, sanitize surfaces, practice good hand hygiene, use personal protective equipment such as masks and gloves, and social distance. They also aimed to cease interactions with friends and family members. One participant stated:

*‘Outside the house, I am definitely keeping my six feet away from anyone.’*

Many participants adopted these standard precautions after gathering information on COVID-19 from multiple sources, and turning to friends, family members, and health providers for advice. One participant recounted:

*‘I have a daughter that’s a nurse. She calls us and coaches us on what to do and not to do. She’s telling us daily what’s going on.’*

Some participants took even more precautions (beyond standard ones), which they felt minimized their risk of transmitting COVID-19. One participant described it as such:

*‘I would spray my whole entire body with Lysol … getting rid of anything that I had on because I was so scared for my grandson.’*

Others declined resources and health-related care services -- such as physical therapy, health aides and physicians’ visits -- in order to decrease transmission risk. One participant explained:

*‘I was without a home health aide for two weeks because I was afraid of getting infected. I had to do everything with one hand because my other hand doesn’t have strength. I couldn’t take a shower.’*

### Theme (3): barriers to social distancing and quarantining

Despite efforts to take precautions, participants felt that several factors made it challenging to social distance, which placed them at an increased risk for COVID-19 transmission. For example, several participants noted that they or their family members were essential workers or healthcare workers, and acknowledged the risks associated with those professions. As one participant expressed it:

*‘My husband, my daughter, they both are essential workers. They both have to go out. They both have to work.’*

In addition to these paid jobs which made social distancing hard, many also served as long-term caregivers to their family members, which further compounded their inability to socially distance. One participant described the experience of caring for her mother, who lived separately:

‘*I take care of everything in the household … She will be in her room while I make sure everything is good, and then I'll just leave. I haven't really touched my mom going on four months, which is hard.’*

Additionally, many felt obligated to care for family members or friends who had become actively infected, despite knowing that they were putting themselves at risk, noting that those individuals would otherwise have no other assistance or support. One participant described such a situation:

‘*When my husband got sick, I couldn’t just leave him. I cared for him. He would stay on the bottom bunk bed, and my son and I on top. That was the only distance for us.*’

Adding to their struggles, many participants described being forced to put themselves at risk in order to run errands for themselves or for their loved ones. One participant noted:

‘*If the doctor asks me to go to an appointment, I need to go. I have to take public transportation to go to Manhattan.*’

Participants also expressed that there was a lack of reliable and accurate information, and at times poor communication from health providers, which made it difficult to understand and trust what precautions to take during the pandemic, including how to isolate. One participant said:

*‘My partner said he felt like he was dying … The doctor told him to stay home and ride it out, and nothing else. He felt very dismissed and unsupported. He ultimately went to the hospital and was hospitalized for four days.’*

Similarly, participants reported uncertainty about the required duration of self-isolation following a potential exposure or positive test, with several explaining that they were told to isolate for months at a time.

### Theme (4): enduring psychological and physical toll of the pandemic

In addition to experiencing barriers to social distancing and self-quarantining, participants also commented that the pandemic – including trying to adhere to these protective measures -- affected their overall wellbeing, particularly their mental health. Many participants referred to feelings of isolation and depression as a function of quarantining from their friends and families. Additionally, they expressed feeling anxious about the pandemic, particularly during quarantining. In several cases, the symptoms of these stressors, including rapid breathing and palpitations, were indistinguishable from those of COVID19, causing further distress, uncertainty, and fear. As one participant explained:

*‘It wouldn’t let me sleep. I was really afraid to. The last time the ambulance came, he told me it was a panic attack.’*

Beyond mental health, participants spoke about the pandemic’s negative impact on their overall physical health, either because of refractory symptoms of COVID-19 or because of deconditioning and food-insecurity during quarantining. One participant noted:

*‘I’d go out a lot, and when they were giving me therapy, I was getting better, I’d go out to play domino at the park. With the pandemic, I stopped going out, and physical therapy shut down. Because of that, everything hurts a lot.’*

Participants also alluded to the financial impact of the pandemic, with many being suddenly and permanently unemployed while simultaneously incurring large unexpected expenses. One participant stated:

*‘The hospital told me to go to a cheap hotel nearby. They charged me $2,000 and I was left without anything. Now, I have no means. There’s no work. I haven't paid rent or electricity in three months.’*

Despite these challenges, participants expressed resilience and discussed how they were adapting their day-to-day routines and mindset to remain strong. One participant summarized:

*‘From here on, keep on fighting and keep on recovering.’*

## Discussion

To our knowledge, this is the first study to describe the experiences of an under-resourced Hispanic community with social distancing and self-quarantining during the COVID-19 pandemic. Our findings suggest that Hispanic adults had substantial knowledge and fears regarding COVID-19, with many of them having been exposed to the virus either directly or through close contacts. Despite attempting to take precautions to minimize their risk, numerous challenges limited their ability to social distance. Their frequent role as essential workers placed them at particularly high risk, as did their simultaneous duties as family caregivers. Additionally, a lack of reliable information, at times from healthcare providers, made it challenging to identify the appropriate precautions to take. Together, their increased risk of exposure coupled with barriers to social distancing, strained the psychological, physical, and financial well-being of this community.

This study expands our understanding of how COVID-19 affects Hispanic communities. Observational data has found that age-adjusted hospitalization rates and deaths are often higher among Hispanics compared to non-Hispanics, and our study sheds light as to why this may be [1, 7, 26]. Beyond age and clinical morbidities, we found that several social determinants of health may be powerful drivers of these observed inequities. First, employment in non-remote essential industries increased participants’ exposure to COVID-19. Second, housing in crowded and multigenerational homes limited their ability to social distance and quarantine when necessary. Indeed, a recent study by Podewils et al. found that among adults with COVID-19, Hispanics reported larger household sizes than non-Hispanics [27]. However, this study did not assess the crowdedness of those households nor the support networks that extend between households. Third, social support networks and providing care as family caregivers may have facilitated the spread of the virus among participants and their loved ones. Future research that accounts for these unique social determinants among Hispanics is needed.

Another key finding in this study was that participants often faced challenges obtaining reliable information regarding COVID-19 despite substantial efforts to do so. We found that beyond the news, participants leveraged their informal social networks, to gain information about COVID-19 and mitigation precautions. They also relied on interactions with the healthcare system, either as healthcare workers or care recipients, to obtain information. However, participants often acknowledged that it was difficult to attain accurate and clear information, noting an abundance of misinformation and poor communication. Future public health interventions should aim to leverage these informal support networks and indirect contacts with the healthcare system to disseminate culturally-tailored COVID-19-related information and messaging that considers the unique barriers to social distancing faced by this population.

Beyond public health messaging, our findings also signal a need to evaluate and address the physical and mental health of Hispanic communities during and after COVID-19. Psychological distress and physical inactivity were common in our sample, particularly as access to certain resources and informal support systems were interrupted. These reports are particularly pertinent given that mental health [28] and sedentary behaviors [29] are each independently associated with obesity, which itself has disproportionately burdened the Hispanic community for decades [30, 31]. In this context, future research should aim to evaluate the longitudinal impact of COVID-19 on the behavioral and physical health of this community. In addition to measuring the collateral impact of the pandemic, policies should be proactive in expanding health insurance coverage and investing in community-engaged collaborations that improve medical and mental health service delivery [32].

Although we used the Health Belief Model to inform our topic guide and the analysis, our four themes do not individually correspond with the four main constructs of the model [17]. Rather, our themes reflect how these constructs are related to each other, and can serve as an example to how others utilize this framework for pandemic-related research. For example, among our participants, the perceived threat of COVID-19 was nearly inseparable from the cue to social distance, and both constructs emerged in one theme (*Fear of Contracting and Transmitting COVID-19*). This adds support to prior findings that individual constructs in the Health Belief Model, particularly cue to action, may combine and interact with each other across different health conditions and behaviors [33, 34]. Similarly, self-efficacy and likelihood of enacting a health behavior both emerged in *Barriers to Enacting Social Distancing and Quarantining*. Participants noted that multiple social determinants of health acted as barriers to social distancing, and while these barriers did not deter them from wanting to engage in practices to reduce transmission, they did make it challenging to perform those practices adequately. Policies aiming to address these barriers to social distancing, including work protections for essential workers and optimizing access to basic goods and services, may influence the likelihood of taking adequate precautions.

### Strengths and limitations

The strengths of our study include our community-engaged approach recruiting a diverse sample of traditionally underrepresented participants. Additionally, interviews were conducted in both Spanish and English. Finally, we analyzed the data using a rigorous thematic analysis which builds upon the health behavior theory. Our study also has several limitations, however. It is a qualitative study that is not generalizable but instead designed to generate hypotheses from the thoroughly explored experiences of participants. Participants were recruited from a single clinical setting, and while it captures a relatively diverse underserved Hispanic population, it does not capture issues pertinent to all subgroups, including those living in rural areas.

## Conclusion

Overall, this under-resourced Hispanic community was substantially affected by the COVID-19 pandemic. Although aware of the risks for contracting COVID-19 and committed to taking appropriate precautions, they faced numerous challenges to social distancing and quarantining. Such challenges took a toll on their physical, emotional, and financial well-being. Our findings suggest that a tailored approach to public health messaging and interventions for pandemic planning are warranted among members in this community. Further research is needed to understand and mitigate the long term physical and psychological consequences of the pandemic among Hispanics.

## Supplementary Information

**Additional file 1.** Interview Topic Guide. The topic guide used in the qualitative interviews.

**Additional file 2.** Code Book. An index of qualitative codes used to analyze the interviews.

## Data Availability

The datasets used and/or analyzed during the current study are available from the corresponding author on reasonable request.

## Abbreviations

COREQ: Consolidated Criteria for Reporting Qualitative Research
COVID-19: Novel coronavirus
PPE: Personal Protective Equipment
US: United States
