# Supplementary material for: Challenges with social distancing during the COVID-19 pandemic among Hispanics in New York City: a qualitative study
Source: BMC Public Health. 2021 Oct 26;21:1946. doi: 10.1186/s12889-021-11939-7 (PMC8547302; doi:10.1186/s12889-021-11939-7)
Supplement: Supplementary file 1 — Additional file 1. Interview Topic Guide. The topic guide used in the qualitative interviews. [file 12889_2021_11939_MOESM1_ESM.pdf]

Additional File 1. Interview Topic Guide

Warm-up and introductory question:

1. What do you know about COVID-19?
2. Please describe your living situation – who do you live with, where, how many rooms does your home or apartment have.

Main questions:

3. Experts advise that people should "social distance." What does this mean to you?
4. Tell me about your experiences with social distancing during the COVID- 19 pandemic.
    a. Probe: What are barriers for you?
    b. Probe: What makes this easier for you?
5. How is social distancing helpful to protecting you against COVID-19?
    a. Probe: How about protecting others?
6. Do you think you are at risk of getting COVID-19? Why?
    a. Probe: What do you think your risk is, compared to other people? Please explain.
7. How are you protecting yourself from getting COVID-19?
8. Who do you live with?
    a. Probe: Are you worried about them getting COVID-19?
    b. Probe: What are you doing to protect them?
    c. Probe: Is there anyone else you are particularly concerned about?
9. Has your job affected your ability to maintain social distance? How so?
    a. Probe: Do you commute? What is it like?
10. Have you received information about how to maintain social distance?
    a. Probe: What kind of information have you received and from where?
11. Do you feel confident about what you should do to maintain social distance?
    a. Probe: Do you have any particular questions about social distancing?

12. Experts also advise that people who have been exposed to COVID-19 or that have symptoms of COVID-19 should self-quarantine. What does this mean to you?

13. If you were asked to self-quarantine, walk me through some of the things that you would do.

    a. Probe: Where would you sleep?

    b. Probe: How would you shower/use the bathroom?

    c. Probe: How would you eat?

    d. Probe: How would you get your medications?

14. What things would make quarantining difficult for you?

15. What things would make quarantining easier?

16. How well do you think you would be able to quarantine during the COVID-19 pandemic?

17. What information would be helpful to have more of during the pandemic?

<u>Closing question</u>

18. Is there anything you want to share that we haven't asked you?
