# Supplementary material for: Challenges with social distancing during the COVID-19 pandemic among Hispanics in New York City: a qualitative study
Source: BMC Public Health. 2021 Oct 26;21:1946. doi: 10.1186/s12889-021-11939-7 (PMC8547302; doi:10.1186/s12889-021-11939-7)
Supplement: Supplementary file 2 — Additional file 2. Code Book. An index of qualitative codes used to analyze the interviews. [file 12889_2021_11939_MOESM2_ESM.pdf]

Additional File 2. Code Book

Codes	Description
Frequent reminders to socially distance	Participant received frequent cues and instructions to socially distance
Fear of COVID-19	Participant expresses fear of contracting or having COVID-19
Known contact	Participant knew someone who had contracted COVID-19
Known contact: Death	Death of a family member or friend from COVID-19
COVID-19 test result	Participants refers to known test results related to COVID-19
Housing	This category refers to codes related to characteristics of the participant's home that relate to COVID-19 risks
Housing: Multi-bedroom	Living in a household with more than one bedroom
Housing: Multi-generational	Living in a household with more than one generation (i.e. parent-child, grandparent-child, grandparent-parent)
Housing: Multi-person	Living in a household with at least one other person
Family in medicine	Participant has a family member who works in healthcare
High perceived risk	Participant feels they are at high risk of contracting COVID-19
Low perceived risk	Participant feels they are at low risk or not at risk of contracting COVID-19
Lack of confidence in taking preventative measures	Participant is not confident in their ability to socially distance, quarantine, or practice other COVID-19-related preventative measures
Confidence in taking preventative measures	Participant is confident in their ability to socially distance, quarantine, or practice other COVID-19-related preventative measures
Precautions to mitigate risks	This category refers to codes related to COVID-19 precautions
Precautions: Keep distance	Staying physically distanced from others
Precautions: Avoid crowds	Avoiding crowds or crowded spaces
Precautions: Avoid people with symptoms	Avoiding people with symptoms
Precautions: Avoid interactions with people from outside the home	Avoiding specific interactions with another person/people outside the home
Precautions: Stay indoors	Attempting to stay indoors
Precautions: Hand hygiene	Hand washing or not touching other things

Precautions: Sanitizing surfaces	Sanitizing a surface (i.e. wiping, lysoling, cleaning)
Precautions: Use of personal protective equipment (PPE)	Specific PPE use (masks, gloves, goggles)
Precautions: Self-monitoring	Self-monitoring of potential COVID-19 signs and/or symptoms
Precautions: Self-quarantine	Participant refers to self-quarantining
Precautions: Working remotely	Working remotely
Information transfer	This category refers to codes related to the participant receiving or giving information related to COVID-19, its risks and related behaviors
Information transfer: Family member	With a family member
Information transfer: Friends	With a friend
Information transfer: Government agency	Through a government agency
Information transfer: TV/news	Through television or televised news, including government press conferences
Information transfer: Health provider	With a health provider
Information transfer: Work	Through work or their occupation
Information transfer: Internet	Through internet searches
Avoid others at home	Participant avoided someone they live with as an exceptional precaution
Disaster preparation	Participant bought extra supplies or strategized for a worst case scenario
Quarantining elsewhere	Participant refers to having to leave one’s home in order to successfully isolate, quarantine or distance from others
Avoid public transportation	Participant refers to the risks associated with public transportation
Drinking fluids	Participant notes the benefits of fluid intake for mitigating the risks COVID-19
Supporting others	Participant cares for or supports someone else, emotionally, or functionally
Ability to isolate at home	Participant is capable of isolating at home
Inability to isolate at home	Participant is not able to successfully isolate at home
Public housing	Participant refers to living in publicly-assisted housing

Others are unhygienic or careless	Participant expresses feelings, opinions, or observations that others are unhygienic (i.e. spitting, dirty, do not practice hand washing)
Healthcare worker	Participant works in a healthcare setting or provides healthcare
Hospital exposure	Participant was in a setting that provides healthcare
Essential worker	Participant is a healthcare worker
Accessing goods and services: Supermarkets	Refers specifically to supermarket, grocery store or required shopping
Accessing goods and services	Participant refers to challenges accessing a good or service, such as food, delivery, or medications
Home schooling	Participants refers to having to home school children
Unreliable information	This category related to transferring inaccurate or unreliable information related to COVID-19
Unreliable information: Poor communication from doctors	Unfavorable communication or interactions with doctors
Unreliable information: Misinformation	Perception that information is not accurate
Unreliable information: Lack of information	Not enough information regarding COVID-19, preventative measures, management, or treatment
Psychological toll	Codes in this category relate to impacts on mental health
Psychological toll: Distress from uncertainty	Distress from uncertainty or not knowing what's happening
Psychological toll: Mental health challenges due to social distancing	Mental health challenges due to social distancing, including anxiety and depression
Lives alone	Participant lives alone
Retired	Participant is retired
Financial strain	Participant refers to experiencing significant financial stress (not having enough money, not being able to pay for rent, buy groceries, etc.)
Financial strain: Not working due to COVID-19	Related to being furloughed or unemployed due to COVID-19
COVID-19 symptoms	Participant refers to symptoms of COVID-19
Physical impact of staying indoors	Participant refers to the consequences of social distancing on personal physical health
Okay to go outside	Participant feeling that going or being outside (of their home) is permissible
Socializing	Participant refers to socializing with friends and family during the pandemic

Coping and self-care	Strategies for coping with any psychological distress and self-care despite the circumstances
Reopening	Participant refers to the ending of stay of home orders
Awaiting vaccine	Participant refers to the anticipated development of the vaccine
Belief in fate and destiny	Participant expresses that future outcomes are destined to occur and outside the hands of the participant, such as "what's meant to happen, is going to happen", "I can't control what's bound to happen," "It's in God's hands"
Belief in cure or treatment	Participant believes in a cure or treatment for COVID-19
